# Sex-specific effects of food supplementation on hibernation performance and reproductive timing in free-ranging common hamsters

**DOI:** 10.1038/s41598-018-31520-4

**Published:** 2018-08-30

**Authors:** Carina Siutz, Margit Valent, Viktoria Ammann, Ariane Niebauer, Eva Millesi

**Affiliations:** Department of Behavioural Biology, University of Vienna, Althanstrasse 14, 1090 Vienna, Austria

## Abstract

Hibernation is characterized by reduced metabolism and body temperature during torpor bouts. Energy reserves available during winter play an important role for hibernation and some species respond to high energy reserves with reduced torpor expression. Common hamsters are food-storing hibernators and females hibernate for shorter periods than males, probably related to larger food stores. In this study, we provided free-ranging common hamsters with sunflower seeds shortly before winter and recorded body temperature using subcutaneously implanted data loggers. We compared hibernation patterns and body mass changes between individuals with and without food supplements and analysed reproductive onset in females. Supplemented males delayed hibernation onset, hibernated for much shorter periods, and emerged in spring with higher body mass than unsupplemented ones. Additional food did not affect hibernation performance in females, but supplemented females emerged earlier and preceded those without food supplements in reproductive onset. Thus, males and females differently responded to food supplementation: access to energy-rich food stores enabled males to shorten the hibernation period and emerge in better body condition, probably enhancing mating opportunities and reproductive success. Females did not alter hibernation patterns, but started to reproduce earlier than unsupplemented individuals, enabling reproductive benefits by an extended breeding period.

## Introduction

Hibernating animals save energy by reducing body temperature and metabolic rate during multiday torpor bouts^1–3^. During winter, they rely on body fat or food stores as energy reserves, which are accumulated before immergence into the hibernacula^4–8^. Accordingly, hibernators show a strict timing of the annual cycle as well as sex- and age-specific sequences in vernal emergence and autumnal immergence^9–15^. Males usually appear above ground in spring before females and shortly thereafter the reproductive period starts with females usually producing one litter per season^12,16–18^. At the end of the active season, adult males usually terminate above ground activity before adult females and, finally, juveniles enter their hibernacula^13^. In addition, some hibernating species show sex differences in torpor expression as males usually start to hibernate later, spend less time in torpor, or terminate hibernation earlier than females^15,19–29^.

In common hamsters (*Cricetus cricetus*), emergence and immergence sequences resemble those of other hibernators^30–32^. Likewise, the reproductive period starts shortly after female emergence in April, but in contrast to other hibernators, female hamsters can produce up to three litters per season, resulting in an extended reproductive period^31,33–35^. Furthermore, common hamsters show exceptional sex differences in hibernation patterns in that adult females delayed hibernation onset for several months and hibernated for shorter periods than males^36^. This could be related to larger food stores due to much more pronounced food caching activities in females compared to males^37^.

The availability of energy reserves was found to affect hibernation performance in several hibernating species and might relate to a cost-benefit trade-off of torpor expression^6^. Despite its apparent advantage of saving energy and reduced predation risk^3,38–42^, hibernation could also have negative effects such as immune depression^43,44^, ischemia^45^, reduced synaptic efficacy^46^, impaired memory retention^47^, oxidative stress^48^, or shortened telomeres^49^. Correspondingly, hibernating individuals with high internal or external energy reserves were found to spend less time in torpor, reduced the depth of torpor, or showed longer euthermic periods^5,27,29,50–52^.

In line with these findings, common hamsters were found to adjust torpor expression in relation to the availability and quality of food stores. We previously demonstrated that hamsters were less likely to hibernate when they had access to food stores compared to individuals facing unpredictable food availability^53^ and, furthermore, hamsters provided with energy-rich food stores almost abandoned deep torpor under laboratory condition^54^. In the field, however, information on the actual quantity and quality of individual food stores is lacking. In this experiment, we therefore provided free-ranging common hamsters with additional food of high energetic content (sunflower seeds) shortly before autumnal immergence and compared the timing of hibernation, torpor patterns, and body mass changes to that of unsupplemented individuals. Among female hamsters, we additionally analysed the timing of reproductive onset (first conception and parturition) in the subsequent season.

## Results

### Immergence and emergence sequences and hibernation patterns

**Figure 1:**
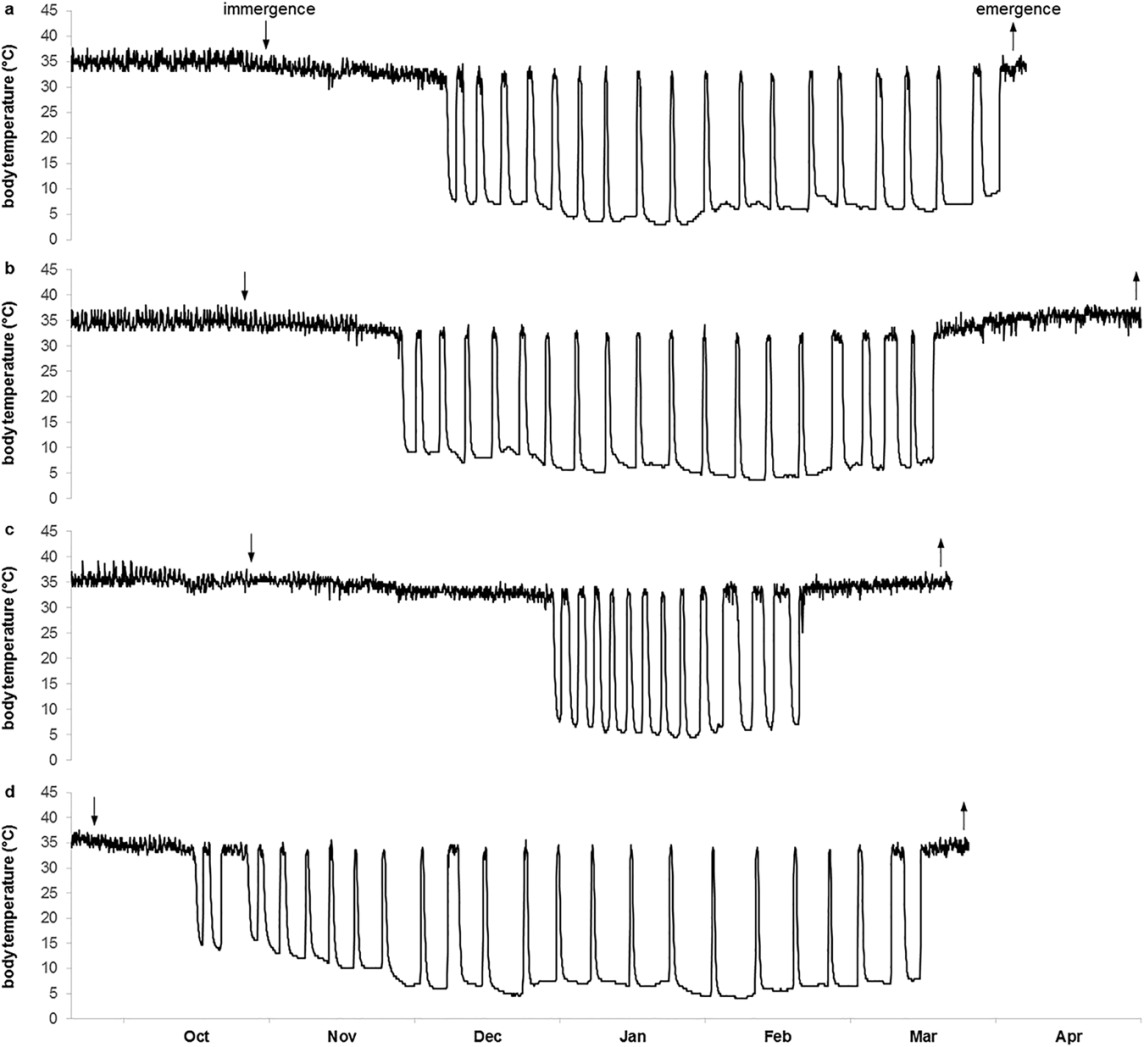
Body temperature patterns of a representative female and male hamster for each group. (**a**) Supplemented female, (**b**) unsupplemented female, (**c**) supplemented male, (**d**) unsupplemented male.

**Figure 2:**
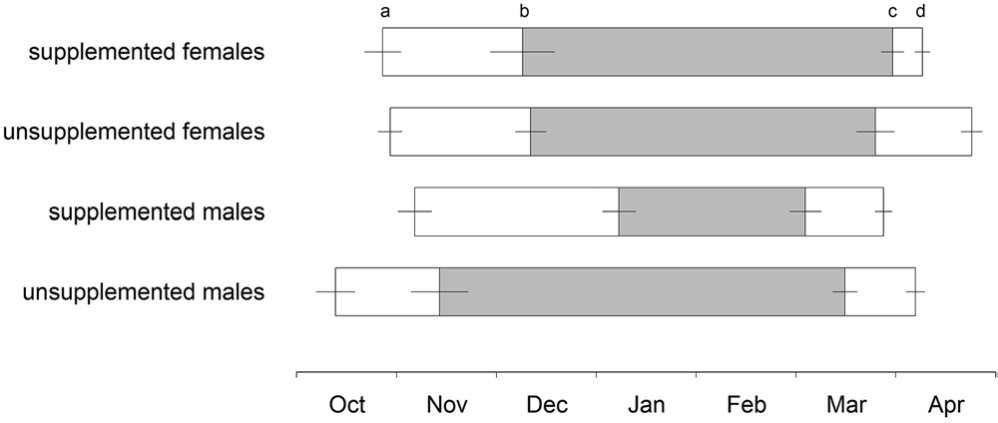
Annual timing and duration of hibernation (grey bars) in unsupplemented and supplemented males and females. (**a**) Indicates immergence into the hibernaculum (date when an individual terminated above-ground activity), (**b**) indicates hibernation onset (date of first torpor bout), **c** indicates hibernation end (date of last torpor bout), and (**d**) indicates vernal emergence from the hibernaculum (date when an individual resumed above-ground activity). Means ± SE; for statistical results and sample sizes see Table 1 and text.

**Figure 3:**
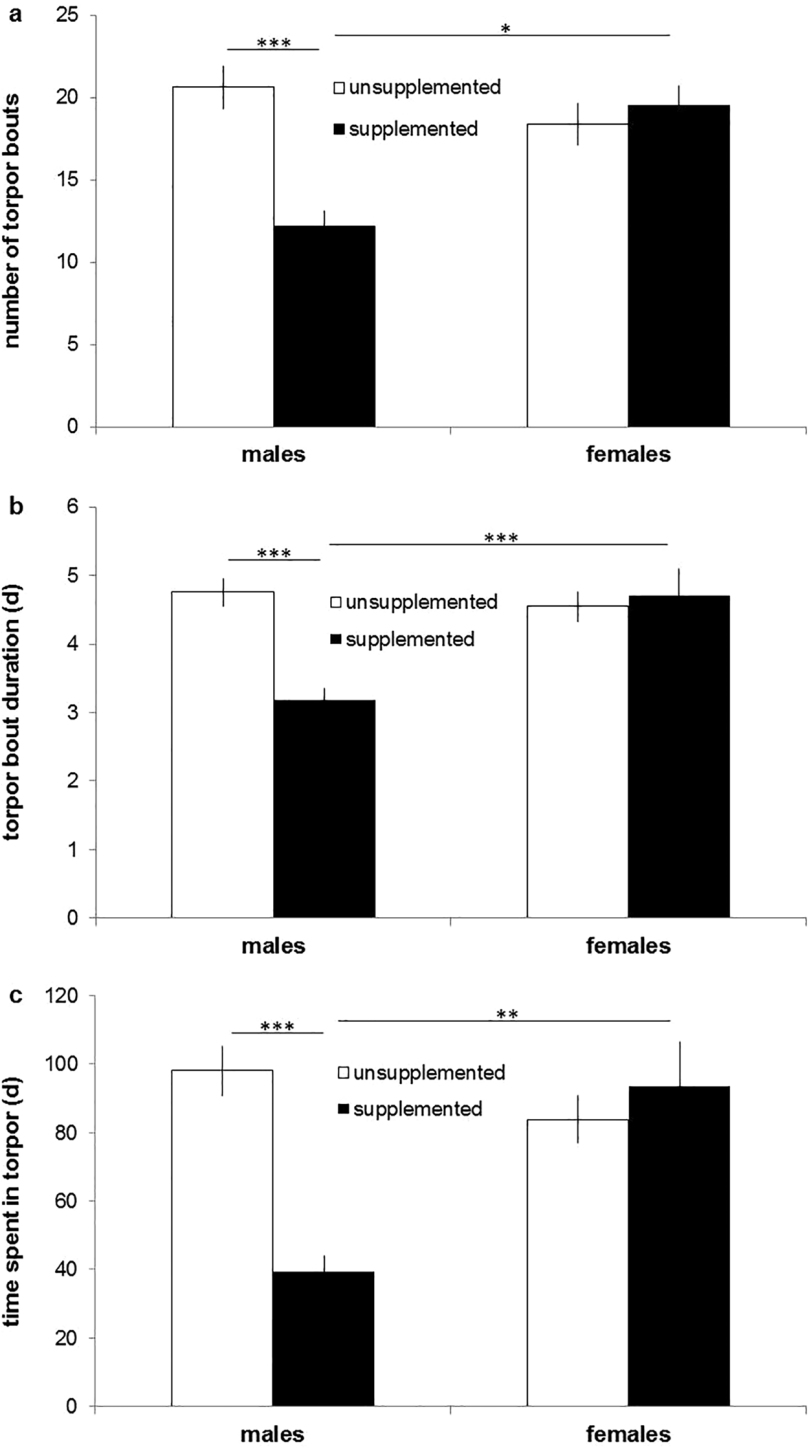
(**a**) Number of torpor bouts, (**b**) mean torpor bout duration, and (**c**) time spent in torpor in unsupplemented and supplemented males and females. Means ± SE; sample size unsupplemented/supplemented: males = 13/6, females = 13/6. *p ≤ 0.05, **p ≤ 0.01, ***p ≤ 0.001.

Body temperature patterns varied between the groups (Fig. 1) and sex-dependent effects of food supplementation were found in most of the parameters (Table 1). Among males, supplemented individuals immerged into their burrows later in the season (F_1,33_ = 9.635, p = 0.008), stayed longer euthermic inside their hibernacula (F_1,33_ = 10.746, p = 0.005), and, correspondingly, delayed hibernation onset (F_1,33_ = 21.847, p < 0.001) compared to unsupplemented ones (Fig. 2). Furthermore, supplemented males showed fewer torpor bouts (F_1,33_ = 16.871, p = 0.001) of shorter durations (χ^2^ = 21.613, p < 0.001), and, accordingly, spent less time in torpor (F_1,33_ = 22.633, p < 0.001) than unsupplemented males (Fig. 3). Minimum and mean body temperature during torpor did not differ between the groups (minimum: supplemented males: 7.8 ± 0.7 °C, unsupplemented males: 8.2 ± 0.5 °C, χ^2^ = 0.300, p > 0.999; mean: supplemented males: 11.1 ± 0.6 °C, unsupplemented males: 10.4 ± 0.4 °C, χ^2^ = 0.626, p = 0.857). Supplemented males terminated hibernation in early March, similar to unsupplemented ones (F_1,33_ = 2.636, p = 0.228), but, due to the delayed onset, hibernated for shorter periods (F_1,33_ = 25.677, p < 0.001; Fig. 2). The duration of post-hibernation euthermy was similar in both groups (F_1,29_ = 0.710, p = 0.812), but supplemented males tended to emerge earlier in spring (F_1,29_ = 5.001, p = 0.066) and, finally, spent less time inside the hibernaculum than unsupplemented ones (F_1,29_ = 14.489, p = 0.001; Fig. 2).

**Table 1:** ANOVA (Type III) table representing effects of sex and group (supplemented/unsupplemented) on annual timing and hibernation performance in common hamsters. Presented values are corrected for age. T_b_…body temperature.

Response variable	Predictor variable	DF	*F* value	*p* value
Immergence date^a^	Sex	1	9.470	0.004
	Group	1	0.006	0.938
	Sex × group	1	5.032	0.032
Prehibernation euthermy (days)^a^	Sex	1	1.829	0.185
	Group	1	0.013	0.910
	Sex × group	1	5.717	0.023
First torpor bout (date)^a^	Sex	1	9.536	0.004
	Group	1	0.021	0.886
	Sex × group	1	11.541	0.002
Number of torpor bouts^a^	Sex	1	2.052	0.161
	Group	1	0.245	0.624
	Sex × group	1	10.534	0.003
Torpor bout duration (hours)^a^	Sex	1	0.261	0.613
	Group	1	0.496	0.486
	Sex × group	1	14.566	0.001
Time spent in torpor (hours)^a^	Sex	1	1.842	0.184
	Group	1	0.672	0.418
	Sex × group	1	15.472	<0.001
Minimum T_b_ during torpor (°C)^a^	Sex	1	2.379	0.133
	Group	1	1.006	0.323
	Sex × group	1	1.189	0.284
Mean T_b_ during torpor (°C)^a^	Sex	1	1.455	0.236
	Group	1	0.365	0.550
	Sex × group	1	0.020	0.888
Last torpor bout (date)^a^	Sex	1	2.903	0.098
	Group	1	0.651	0.426
	Sex × group	1	2.941	0.096
Hibernation duration (days)^a^	Sex	1	3.049	0.090
	Group	1	0.354	0.556
	Sex × group	1	15.943	<0.001
Post-hibernation euthermy (days)^b^	Sex	1	1.340	0.257
	Group	1	9.513	0.005
	Sex × group	1	7.857	0.009
Emergence date^b^	Sex	1	22.887	<0.001
	Group	1	10.744	0.003
	Sex × group	1	0.642	0.429
Time inside hibernaculum (days)^b^	Sex	1	0.003	0.960
	Group	1	2.501	0.125
	Sex × group	1	2.279	0.142

^a^Sample size supplemented/unsupplemented: males = 6/13, females = 6/13.

^b^Sample size supplemented/unsupplemented: males = 6/12, females = 6/10.

Among females, we found neither differences in timing of immergence nor hibernation performance between supplemented and unsupplemented individuals (p > 0.628 in all cases; Table 1, Figs 2 and 3; minimum body temperature: supplemented females: 8.2 ± 1 °C, unsupplemented females: 7.3 ± 0.4 °C; mean body temperature: supplemented females: 10.4 ± 0.9 °C, unsupplemented females: 9.8 ± 0.5 °C). Supplemented females, however, had a shorter duration of post-hibernation euthermy (F_1,29_ = 9.513, p = 0.009) and emerged from their hibernacula earlier in spring (F_1,29_ = 10.744, p = 0.005) than unsupplemented females (Table 1, Fig. 2). The time inside the hibernaculum was similar in females with and without food supplements (F_1,29_ = 2.501, p = 0.249).

### Sex differences among supplemented individuals

Supplemented males and females immerged into their hibernacula and started to hibernate at similar dates (immergence: F_1,33_ = 0.363, p > 0.99; hibernation onset: F_1,33_ = 3.881, p = 0.115; Table 1, Fig. 2). Males, however, terminated hibernation earlier (F_1,33_ = 10.174, p = 0.006) and hibernated for shorter periods compared to females (F_1,33_ = 12.835, p = 0.002; Fig. 2), showed fewer and shorter torpor bouts (number: F_1,33_ = 8.429, p = 0.013; duration: χ^2^ = 17.575, p < 0.001), and, correspondingly, spent less time in torpor than females (F_1,33_ = 14.239, p = 0.001; Fig. 3). Minimum and mean body temperature did not differ between the sexes (minimum: χ^2^ = 0.075, p > 0.99; mean: χ^2^ = 0.933, p = 0.668). Finally, males showed a longer euthermic phase after hibernation (F_1,29_ = 6.541, p = 0.032) and emerged earlier from their burrows than females (F_1,29_ = 6.669, p = 0.030; Fig. 2). The time spent inside the hibernaculum, however, was similar in both sexes (F_1,29_ = 3.531, p = 0.141; Fig. 2).

### Body condition

**Figure 4:**
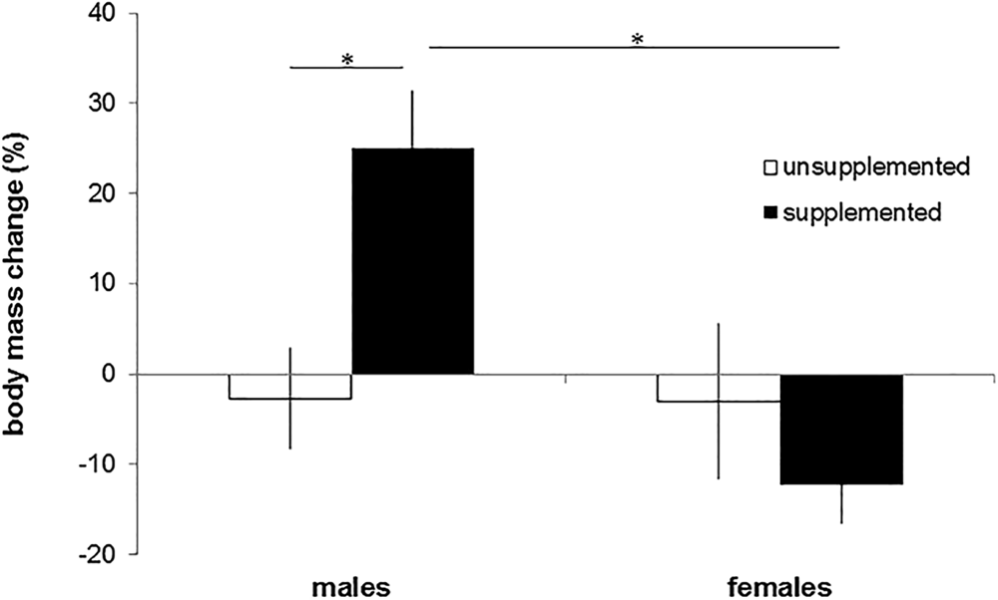
Body mass change (%) in unsupplemented and supplemented males and females. Means ± SE; sample size unsupplemented/supplemented: males = 12/6, females = 9/6. *p ≤ 0.05.

Body mass at immergence was similar in supplemented and unsupplemented individuals of both sexes (supplemented males: 339.3 ± 25.6 g, n = 6, unsupplemented males: 333.9 ± 16.3 g, n = 13, F_1,33_ = 0.210, p > 0.99; supplemented females: 294.7 ± 16.7 g, n = 6, unsupplemented females: 330.2 ± 23 g, n = 13, F_1,33_ = 3.847, p = 0.117). In spring, supplemented males emerged with higher body mass than unsupplemented males (supplemented males: 419.7 ± 26.1 g, n = 6, unsupplemented males: 319.9 ± 18.8 g, n = 12, F_1,28_ = 11.140, p = 0.005), while no differences were found among females (supplemented females: 259.5 ± 21.9 g, n = 6, unsupplemented females: 317.2 ± 23.1 g, n = 9, F_1,28_ = 4.028, p = 0.109). Body mass change over winter significantly differed between supplemented and unsupplemented males (F_1,28_ = 8.565, p = 0.014) as supplemented males increased their body mass during winter, while the others lost mass (Fig. 4). In females, however, body mass loss was similar in both groups (F_1,28_ = 0.615, p = 0.880; Fig. 4).

### Female subsequent reproductive timing

**Figure 5:**
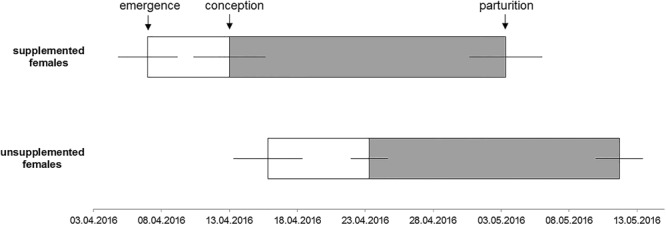
Timing of reproduction after vernal emergence as indicated by first conception and first parturition date in the respective season in unsupplemented and supplemented females. Means ± SE; sample size (*p*-values) unsupplemented/supplemented: emergence = 13/6 (p = 0.018), conception = 14/6 (p = 0.009), parturition = 13/6 (p = 0.027). For detailed statistical results see text.

When comparing females from the same season exclusively, we again found that supplemented females emerged earlier in spring than unsupplemented ones (t = 2.646, p = 0.018; Fig. 5). Females of both groups started to reproduce about one week after vernal emergence (t = −0.177, p = 0.862; Fig. 5). Consequently, females provided with additional food mated earlier in the season (t = 3.457, p = 0.009) and also gave birth to their first litter earlier than those without food supplements (t = 2.623, p = 0.027; Fig. 5).

## Discussion

The provision of energy-rich food shortly before winter produced sex-specific effects on annual timing and hibernation performance in free-ranging common hamsters. Supplemented males immerged into their hibernacula later in the season than unsupplemented ones. In common hamsters, males usually terminate above-ground activity before females, but by extending the active season, supplemented males immerged at a similar time as females. This could have enabled these males to detect and monitor female hibernacula already in autumn, which would be advantageous in the subsequent spring because they could concentrate their activity on areas where oestrous females most likely emerge^13^. Behavioural observations supported this assumption as the first mating events in spring involved supplemented males. However, due to the large home range sizes of males during the breeding period we were unable to compare mating success of supplemented and unsupplemented individuals. Autumnal weather conditions were relatively mild but similar to previous seasons, indicating that this did not account for the delayed immergence. Furthermore, we found no differences in immergence body mass between supplemented and unsupplemented individuals indicating that the provided food was not consumed prior to immergence. After immergence, supplemented males showed an extended euthermic period inside the hibernaculum, resulting in a delayed hibernation onset and strongly reduced hibernation durations compared to unsupplemented individuals. Accordingly, males provided with additional food showed fewer torpor bouts of shorter duration and spent less time in torpor than unsupplemented ones. Given the remarkably long duration of prehibernation euthermy, males had to consume at least parts of their food stores during this time and expressed torpor only during the coldest winter months (mainly January/February) when this energy-saving state was probably most beneficial. By reducing not only the hibernation period but also the duration of deep torpor bouts, supplemented males probably were able to minimize potential costs of torpor as negative effects might become more intense with increasing torpor bout length. Moreover, supplemented males could benefit from shorter torpor bouts as improved environmental conditions towards the end of the winter period can be detected more quickly. This would be particularly advantageous for the timing of vernal emergence as indicated by an earlier start of the active season in supplemented males compared to those without additional food. We have no information on when the food was consumed in the burrow, but considering the extended prehibernation phase in supplemented males it is likely that they started to hibernate in an improved body condition and with higher body fat proportion compared to autumnal immergence and thus, could afford shorter torpor bout durations during the coldest period in winter.

We previously demonstrated that not only the quantity but particularly the quality of food stores is crucial for hibernation performance. Hamsters with access to large food stores and high sunflower seeds intake virtually abandoned deep torpor under laboratory conditions^54^. In the presented study, we enlarged and qualitatively improved the natural food stores of both males and females, but only males responded to food supplementation by shortening hibernation. Sunflower seeds resemble food items stored by common hamsters at our study site (e.g., beechnuts, hazelnuts, acorn or black walnuts) in the caloric value and nutrient composition (including fat content)^55^. The reduced torpor expression in supplemented males, therefore, unlikely resulted from sunflower seeds *per se*, because in this case one would expect a shortened hibernation period in supplemented females as well. Our results rather indicate that male common hamsters, which usually have limited access to external energy reserves as indicated by less pronounced food caching activities^37^, minimize hibernation when sufficient, energy-rich food is available.

Furthermore, access to high-quality food stores not only enabled supplemented males to emerge about two weeks earlier in spring than unsupplemented ones but, particularly, to gain body mass over winter, which could be a considerable advantage regarding reproductive success. Common hamsters have a promiscuous mating system and males face a high intrasexual competition, particularly at the onset of the mating period shortly after female emergence^56^. Furthermore, males usually have scrotal testes at vernal emergence, but testes size continuously increases until shortly after mating onset^57^. Early emerging males, therefore, might be able to complete testes growth prior to mating onset and, particularly, a better body condition in spring would enhance their competitive potential^57,58^ and be beneficial for enduring the long and exhausting mating period, lasting until August, which, ultimately, might increase their reproductive success.

Interestingly, food supplementation did not affect the timing of autumnal immergence and patterns of torpor expression in females, indicating that the additional food was not used to shorten the hibernation period. As a result, food supplementation largely reversed the previously documented sex differences in hibernation performance^36^ as now males hibernated for shorter periods, expressed fewer and shorter torpor bouts, and spent less time in torpor than females. Supplemented females, however, strongly shortened the duration of post-hibernation euthermy and emerged earlier in spring than unsupplemented ones. Moreover, females provided with additional food preceded those without supplements in the date of first conception and parturition. This temporal advantage is of major importance as female common hamsters can produce up to 3 litters per season and it has been previously shown that the earlier a female emerged and started to reproduce in spring, the more litters and more offspring were produced^33^, which was also found in all seasons investigated at our study site to date (Siutz *et al*., unpublished data). Thus, females seem to have used the supplemented food for an earlier start of the active season and reproductive onset, most likely resulting in a higher seasonal reproductive output. This is supported by similar emergence body mass and body mass losses over winter in supplemented and unsupplemented females, indicating that supplemented individuals conserved at least parts of the provided food. In contrast to males, which frequently change their burrows during the active season, female hamsters are more philopatric and could, therefore, benefit from remaining food stores as energy source during gestation and lactation, allowing them to decrease above-ground activity and, hence, exposure to predation risk, as well as to have access to high-quality food when seasonally not available.

A limiting factor of this study might be that hibernation data of supplemented and unsupplemented individuals were not collected in the same year. Although annual effects cannot be completely excluded, it is unlikely that our findings resulted from seasonal variations in environmental or climatic conditions. First, we could already show in unsupplemented individuals that timing of hibernation and hibernation performance itself were not affected by different years^36^. Although immergence and emergence sequences follow sex- and age-specific patterns, annual variations within these groups are usually low, which is not surprising as the seasonal timing of hibernation is mainly triggered by an endogenous circannual clock^3^. Since in our season of food supplementation, ambient temperatures during autumn, winter, and spring as well as food availability and snow cover were similar to the previous ones, it seems unlikely that environmental or climatic conditions caused the differing hibernation patterns in supplemented hamsters. Second, body temperature during torpor usually drops close to ambient temperature inside the hibernaculum^2^. We found, however, no differences in minimum and mean body temperature during torpor between supplemented and unsupplemented individuals, indicating that ambient temperatures inside the hibernaculum were similar in all years.

In conclusion, our results highlight sex-specific responses to food supplementation as males and females differently used additional food, which might reflect the sex-specific benefits regarding reproductive success, i.e. body condition in males and vernal timing in females. Moreover, the availability of high-energy food could increase an individual’s subsequent reproductive output, which would be of particular importance in this endangered species. Common hamsters face an ongoing population decline, particularly in Western Europe, and are strictly protected by the Bern Convention (Appendix II) and the Fauna-Flora-Habitat (Appendix IV) directives. Several reasons for the decline have been suggested including high overwinter mortality caused by insufficient food stores and, recently, a considerable decline in reproductive output was documented^32,59,60^. Our findings, therefore, could provide valuable information for agricultural management and reintroduction projects as enabling access to sufficient food of high quality at critical time periods could have the potential to counteract the population decline in common hamsters.

## Methods

### Ethical statement

All procedures performed on animals were carried out in accordance with EU guidelines for the protection of animals used for scientific purposes (Directive 2010/63/EU) and were approved by the ethics committee of the Faculty of Life Sciences, University of Vienna (2015-010), the Austrian Federal Ministry of Education, Science and Research (GZ: BMWFW-66.006/0013-WF/V/3b/2015), and the City of Vienna (MA22-2484/10, MA22-310/11).

### Field techniques

We investigated a free-ranging population of common hamsters inhabiting urban areas in southern Vienna by applying capture-mark-recapture techniques during the active season (March/April–October/November 2015) using Tomahawk live traps baited with peanut butter. Hamsters were examined by using cone-shaped cotton sacks, laterally equipped with Velcro fasteners, allowing animal handling without anaesthesia. We subcutaneously implanted a transponder (Data Mars) and fur-marked each individual in different, distinguishable patterns. Sex and age, classified as adult (hibernated at least once) or juvenile (born in the current season) were determined and body mass (±1 g) was recorded at each capture. Further details of our field methods are described elsewhere^33,36,56^. We monitored all hamsters until trapping and observation failure in autumn, indicating immergence into the hibernaculum, and defined the date when the individual was trapped or observed for the last time during the pre-hibernation period as immergence date. To confirm that an individual terminated above-ground activity, we plugged the burrows with leaves to detect potential activity and monitored the burrow entrances at daily intervals. Immergence body mass referred to the individual’s mass recorded within 1 week before its immergence into the hibernaculum. During winter, we checked the burrows (open/closed) weekly, but detected no signs of activity outside the burrow until spring. Beginning in early March, the burrow monitoring was done at daily intervals and active individuals were recaptured. The date when an individual was observed above-ground or trapped for the first time was defined as emergence date, which coincided with the day when an individual had removed its burrow plug. Emergence body mass corresponded to an individual’s weight measured within 1 week after emergence from the hibernaculum. The percentage of body mass change over winter was defined as the difference between an individual’s immergence and emergence body mass.

### Food supplementation

The hamsters were supplemented in late autumn shortly before immergence into the hibernaculum (September/October) to ensure that the food was used for the winter period. We placed 500 g sunflower seeds (Dehner Natura, Dehner GmbH, Germany) in front of an individual’s hibernaculum and continuously observed the burrow until the hamster had carried all seeds inside. Thus, no other than the focal individual collected the seeds. Sunflower seeds were chosen because of their storability and high energetic content (2.45 MJ/100 g, 51.5 g total fat/100 g)^55^. The caloric value and nutrient composition of sunflower seeds is similar to the natural winter diet of common hamsters at our study site^55^, which consists of, e.g., beechnuts, hazelnuts, acorn, and black walnuts^61^. In addition, hamsters in urban habitats to some extent have access to sunflower seeds (which either are naturally growing or comprised in birdseeds that are occasionally provided by humans) and due to their relatively small size they can be cached more quickly compared to, e.g., hazelnuts, by that facilitating our experimental procedure.

As common hamsters are strictly protected by the Bern Convention (Appendix II) and the Fauna-Flora-Habitat (Appendix IV) directives, we were permitted to implant temperature data loggers (see methods section ‘hibernation patterns’) in 20 individuals (GZ BMWFW-66.006/0013-WF/V/3b/2015). The monitoring of individuals throughout the active season and particularly in autumn allowed us to identify the hibernacula of 18 hamsters. Due to this relatively small sample size as well as considering potential overwinter mortality, all 18 individuals were supplemented. Among these individuals were also young of the year, but all of them were born in spring, had a body mass within the range of adults in autumn, and had completed sexual maturation as indicated by developed, scrotal testes (in case of males) and an opened vagina (in case of females). These individuals were defined as subadult. In the subsequent spring, all individuals sampled in this study were reproductively active. Unsupplemented individuals with available hibernation data from previous years served as control group (7 adult females, 6 subadult females, 5 adult males, 8 subadult males). This data set was already analysed and published^36^ and showed that hibernation performance and seasonal timing were similar in these years so that annual effects can be excluded. Furthermore, we documented climatic conditions (ambient temperature (T_a_) and snow cover) in each year and found no variations between years (T_a_ autumn: 13.7 ± 0.3 °C, F_3,103_ = 0.289, p = 0.833; T_a_ winter: 2.9 ± 0.8 °C, F_3,12_ = 0.798, p = 0.519; T_a_ spring: 10.6 ± 0.3 °C, F_3,99_ = 0.157, p = 0.925; date of first snow cover: 24.12 ± 19 d; date of last snow cover: 25.02. ± 16 d). Moreover, the season of food supplementation did not differ from previous ones (Student’s *t* tests, T_a_ autumn: unsupplemented: 13.8 ± 0.4 °C, supplemented: 13.4 ± 0.7 °C, p = 0.589; T_a_ winter: unsupplemented: 2.4 ± 0.8 °C, supplemented: 4.5 ± 1.7 °C, p = 0.238; T_a_ spring: unsupplemented: 10.7 ± 0.4 °C, supplemented: 10.4 ± 0.7 °C, p = 0.707).

### Hibernation patterns

Body temperature during winter was recorded at 90-min intervals using temperature data loggers (iButtons, DS1922L-F5#, range: −40 °C to + 85 °C, accuracy: ± 0.5 °C, Maxim Integrated Products International, Dublin, Ireland). Immediately after trapping in the morning, the hamsters were transported to a veterinary clinic (~20 min) where the iButtons (coated in Elvax ethylene vinyl acetate resins, DuPont, and paraffin; gas-sterilised; potted mass: ~4.5 g) were implanted subcutaneously in the neck region (dorsal, between the scapulae) under inhalation anaesthesia (isoflurane) and Metacam (0.2 mg/kg body mass) was administered as analgesic before surgery. As soon as the individuals had recovered from anaesthesia (within 5–10 min), we returned them to the field site and released them in front of their burrows (1–2 h after trapping), which were blocked in the meantime to prevent other hamsters from entering the burrows. In spring, iButtons were removed using the same technique, which has proved successful in this species^36,53^ and was also applied to all individuals used in the analyses as control (unsupplemented) group. We could recover iButtons of 12 supplemented individuals (4 adult females, 2 subadult females, 2 adult males, 4 subadult males).

Torpor bouts were defined as periods of reduced body temperature for longer than 24 h, beginning from the sampling interval when body temperature decreased below 30 °C to at least 15 °C until it had reached 30 °C again. To characterize hibernation patterns, the following parameters were determined: hibernation onset (date of the first torpor bout onset), duration of prehibernation euthermy (days from immergence until hibernation onset), number of torpor bouts, torpor bout duration (calculated in hours, expressed as days), time spent in torpor (total duration of all torpor bouts; calculated in hours, expressed as days), minimum body temperature (lowest value of body temperature during a torpor bout), mean body temperature (beginning from the sampling interval when body temperature decreased below 30 °C until it had reached 30 °C again), hibernation end (date of the last torpor bout termination), hibernation duration (days from the onset of the first to the termination of the last torpor bout), duration of post-hibernation euthermy (days from hibernation end until emergence), and time spent inside the hibernaculum (days from immergence to emergence).

### Female subsequent reproductive timing

After vernal emergence, we were able to monitor all six supplemented females until the end of their first lactation period (late June). In addition, 14 unsupplemented females (without hibernation data) could be monitored during the same period. Some of these individuals were already trapped in the previous autumn, but immerged into their hibernacula before we could implant temperature data loggers. We, therefore, were able to compare reproductive timing via the dates of conception and parturition in supplemented and unsupplemented females of the same season. All individuals were individually fur-marked for distant recognition and observed at daily intervals. In addition, we captured females at weekly intervals and recorded body mass and teat size based on a 3-point-scale from small (1) to swollen with milk remains^33,56^. The onset of reproduction (conception date) was defined as the date of observed copulations if followed by cumulative body mass gain indicating gestation. Parturition was defined as distinct body mass loss followed by strongly increasing teat size, partly with milk rests, indicating lactation. By assuming 17–20 d of gestation^62^, we intensified trapping around the expected day of parturition, which allowed us to determine the date of parturition with an accuracy of ±2 d. As the exact date of litter emergence could only be detected in two supplemented females, we were not able to compare this parameter between supplemented and unsupplemented females.

### Statistics

Statistical analyses were performed in R^63^ by using the packages ‘car’^64^ for linear models, ‘nlme’^65^ for linear mixed models (LMEs), and ‘phia’^66^ for post-hoc analyses of significant interaction effects. We applied LMEs for the parameters torpor bout duration, minimum body temperature, and mean body temperature and included the parameters sex, group (supplemented/unsupplemented), and their interaction as fixed effects and individual identity as a random effect to correct for repeated measurements. All other parameters (Table 1) were analysed using linear models, including the parameters sex, group (supplemented/unsupplemented), and their interaction as predictor variables. In addition, we corrected for differences in age by including the parameter age (adult/subadult) as predictor variable in each model, but omitted its interactions with the parameters sex and group due to small sample sizes among supplemented individuals. To simplify the presentation of these results, we omitted the parameter age in Table 1, but all values presented are corrected for age. We further only stated sex differences among supplemented individuals in the results section since sex differences among unsupplemented individuals were already published^36^. Model residuals were tested for normality using Shapiro–Wilk tests and for homoscedasticity by Levene-tests. Not normally distributed parameters (immergence body mass and duration of post-hibernation euthermy) were transformed by applying the natural logarithm. Statistics were obtained from ANOVA (Type III) tables; all post-hoc analyses were Bonferroni-corrected. For comparisons of subsequent reproductive timing (dates of emergence, conception, and parturition) between supplemented and unsupplemented females we applied Student’s *t* tests. Significance level was set at *p* ≤ 0.05. Results are presented as means ± s.e.m.

## Data Availability

The datasets generated and/or analysed during the current study are available from the corresponding author on reasonable request.
